# Supplementary material for: Bioactive Compounds in Moringa oleifera Lam. Leaves Inhibit the Pro-Inflammatory Mediators in Lipopolysaccharide-Induced Human Monocyte-Derived Macrophages
Source: Molecules. 2020 Jan 2;25(1):191. doi: 10.3390/molecules25010191 (PMC6982846; doi:10.3390/molecules25010191)
Supplement: Supplementary file 1 [file molecules-25-00191-s001.pdf]

SUPPLEMENT FIGURES

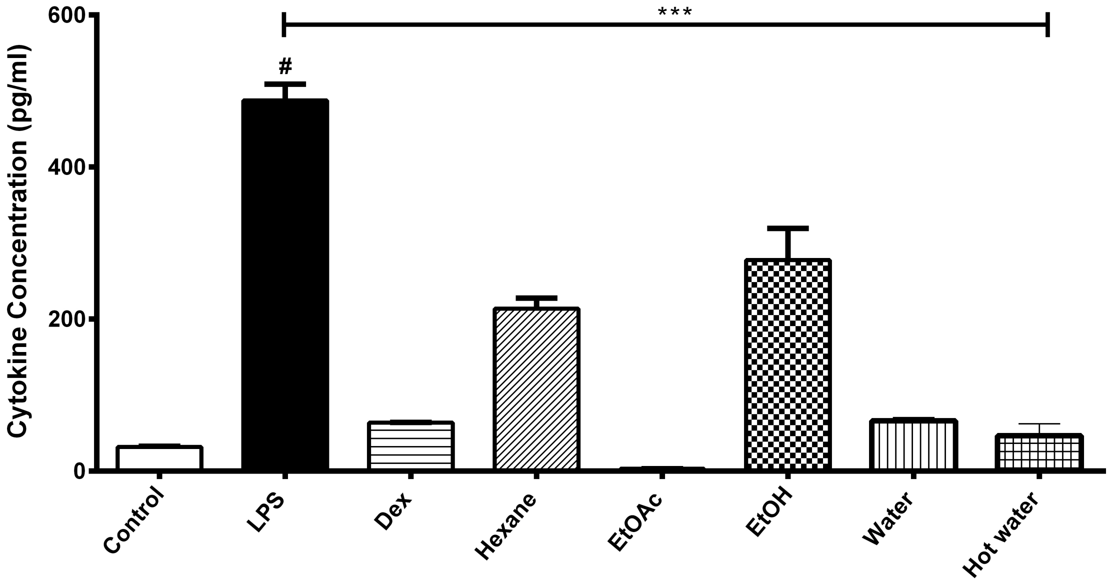

Figure S1: Effect of various MO leaves crude extracts on pro-inflammatory cytokines levels in LPS-induced MDM. Data are presented as means ± SEM. #p < 0.05 compared to control, ***p < 0.05, compared to LPS. Control: Untreated MDM; LPS: Lipopolysaccharide-stimulated MDM; Dex: Dexamethasone; EtOAc: Crude ethyl acetate; EtOH: Crude ethanol

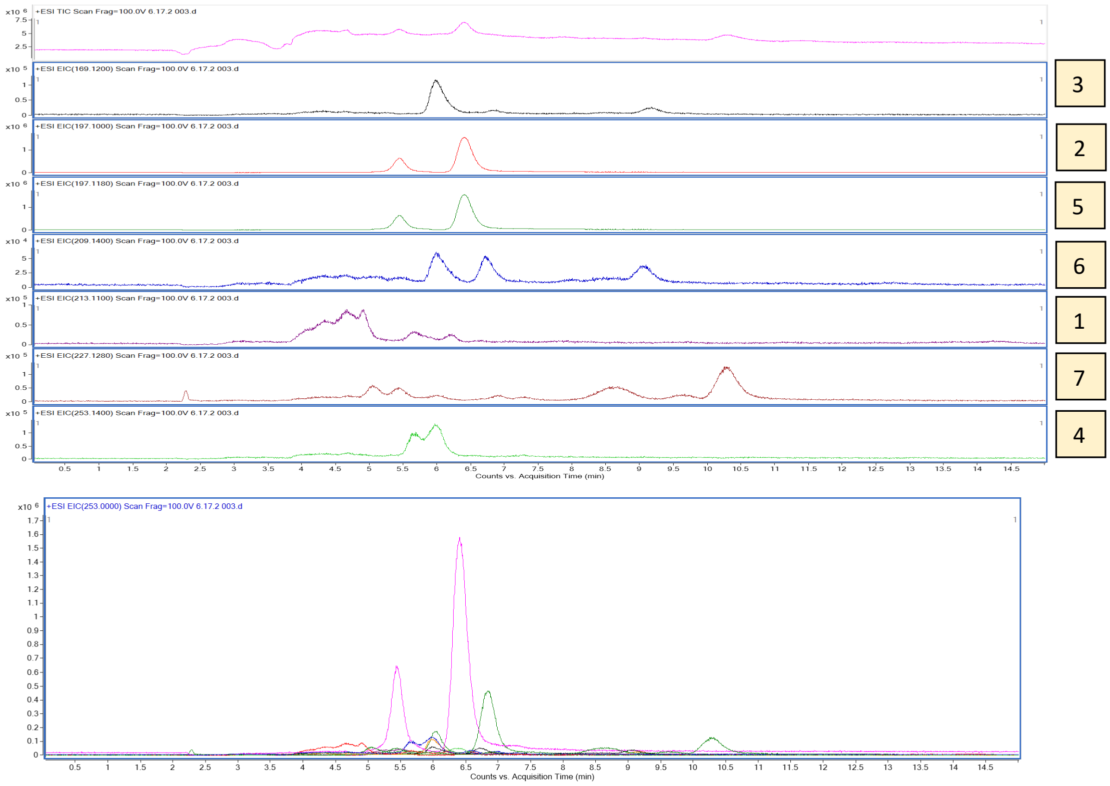

Figure S2: Overlays of LC-MS chromatograms of MO subfraction 6.17.2 with active compounds no.1-7

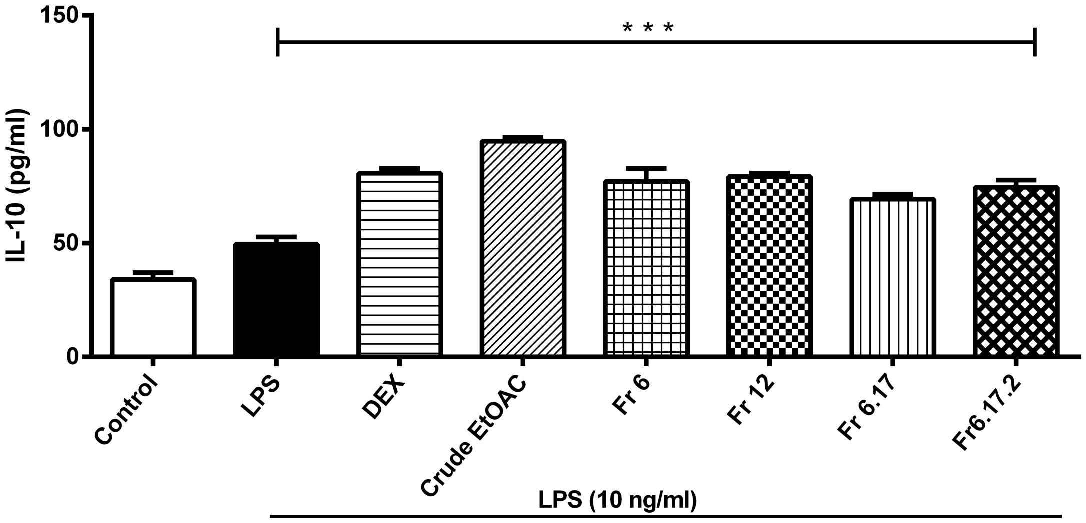

Figure S3: Effect of various MO leaves crude extracts on IL-10 anti-inflammatory cytokine level in LPS-induced MDM. Data are presented as means ± SEM. #$p < 0.001$ compared to control, ***$p < 0.05$, compared to LPS. Control: Untreated MDM; LPS: Lipopolysaccharide-stimulated MDM; Dex: Dexamethasone; Crude EtOAc: Crude ethyl acetate; Fr: Fraction
